# Associations of the serum *n*-6 PUFA concentrations with exercise-induced myocardial ischaemia in middle-aged and older men

**DOI:** 10.1017/S0007114524001971

**Published:** 2024-12-14

**Authors:** Haleh Esmaili, Behnam Tajik, Tomi-Pekka Tuomainen, Sudhir Kurl, Jukka T. Salonen, Jyrki K. Virtanen

**Affiliations:** 1 University of Eastern Finland, Kuopio Campus, Institute of Public Health and Clinical Nutrition, Kuopio, Finland; 2 University of Helsinki, The Faculty of Medicine, Department of Public Health, Helsinki, Finland; 3 MAS-Metabolic Analytical Services Oy, Helsinki, Finland

**Keywords:** *n*-6 PUFA, Exercise, Myocardial ischaemia, Population study

## Abstract

*n*-6 PUFA, especially linoleic acid (LA) but also arachidonic acid (AA), have been inversely associated with CHD. However, mechanisms underlying these associations are not fully known. We investigated the associations of the serum concentrations of total *n*-6 PUFA, LA, AA, *γ*-linolenic acid (GLA) and dihomo-*γ*-linolenic acid (DGLA), with the odds of myocardial ischaemia during exercise, a predictor of future cardiac events. A total of 1871 men without a history of CHD from the Kuopio Ischaemic Heart Disease Risk Factor Study (KIHD) aged 42–60 years were included. All participants performed a maximal symptom-limited exercise stress test, using an electrically braked bicycle ergometer. Multivariable-adjusted logistic regression was used to assess the OR for exercise-induced myocardial ischaemia in quartiles of the serum *n*-6 PUFA concentrations. After multivariable adjustment, men in the highest *v*. the lowest serum AA concentration had 50 % lower odds for exercise-induced myocardial ischaemia (OR 0·50, 95 % CI 0·34, 0·76; *P*-trend across quartiles < 0·001). For the other PUFA, the OR (95 % CI) were 1·00 (0·69, 1·46; *P*-trend = 0·89) for LA, 1·07 (0·75, 1·53; *P*-trend = 0·40) for GLA and 0·74 (0·51, 1·07; *P*-trend = 0·16) for DGLA. Among the *n*-6 PUFA, higher serum concentration of AA was associated with lower odds for myocardial ischaemia during an exercise test in middle-aged and older men. This may provide one mechanism for the previously observed possible cardioprotective properties of AA. Our findings also suggest that *n*-6 PUFA should not be considered as one homogenous group.

CHD is a leading cause of disability and mortality, especially among the elderly^(1)^. It has been shown that myocardial ischaemia is a valuable predictor of CHD and cardiac mortality^(2,3)^. Myocardial ischaemia is defined as an imbalance between myocardial oxygen supply and demand due to blood flow reduction through coronary arteries, leading to cardiac and microvascular dysfunction, myocardial infarction and sudden death^(4)^. The optimal clinical method to assess early plasma changes related to myocardial ischaemia is the cardiac stress test^(5)^, as indicated by transient ST-segment depression in electrocardiogram (ECG) during a stress or exercise test^(6)^.

Previously in the Kuopio Ischaemic Heart Disease Risk Factor Study (KIHD), higher exercise-induced myocardial ischaemia has been related to an increased risk of CHD and sudden death^(6,7)^. Moreover, it has been shown that exercise-induced myocardial ischaemia can predict the risk of atherosclerosis and the prognosis of future cardiac events by influencing the sub-endocardial blood flow and myocardial oxygen consumption^(8,9)^.

Among dietary factors, the cardiovascular benefits of the *n*-3 PUFA, especially the long-chain *n*-3 PUFA from fish, have been quite well established^(10)^. However, the possible benefits of the *n*-6 PUFA on CVD risk have been more debated, although the *n*-6 PUFA, especially the main *n*-6 PUFA linoleic acid (LA, 18:2*n*-6), has been associated with lower risk of CHD^(11)^ and CVD^(12)^. This also seems to be independent of the exposure to the *n*-3 PUFA^(12)^.

LA and the *n*-3 PUFA *α*-linolenic acid (ALA; 18:3*n*-3) are the two essential fatty acids for humans because they cannot be synthesised in the body. They can be used as the precursors for synthesis of longer-chain *n*-6 and *n*-3 PUFA, respectively. LA, which in diet is found mainly in vegetable oils, nuts and seeds^(13)^, can be endogenously converted to other *n*-6 PUFA including *γ*-linolenic acid (GLA, 18:3*n*-6), dihomo-*γ*-linolenic acid (DGLA; 20:3*n*-6) and arachidonic acid (AA, 20:4*n*-6). AA is also found in some foods such as eggs and meat^(13)^, and it has been associated with lower risk of CHD and CVD in some studies^(12,14)^. There are very few dietary sources of GLA and DGLA, so their concentrations reflect endogenous processes. In addition to diet, also other factors affect the concentrations and the synthesis of the longer-chain *n*-3 and *n*-6 PUFA in the body, including nutritional status, certain diseases, genetics, ageing and lifestyle factors^(15–17)^.

Although there are potential mechanisms that have been proposed to explain the beneficial associations of LA with CVD risk^(11,18)^, especially the beneficial impact on serum LDL-cholesterol reduction, little is known about the associations of LA and the other *n*-6 PUFA with myocardial ischaemia during exercise. Therefore, the aim of our study was to elucidate the associations of the serum *n*-6 PUFA concentrations with exercise-induced myocardial ischaemia among middle-aged and older men from the KIHD cohort.

## Materials and methods

### Study population

We performed a cross-sectional analysis among the participants from the KIHD cohort, using data from the baseline analyses from 1984 to 1989. The KIHD is a prospective population-based study designed to investigate risk factors for CVD, carotid atherosclerosis and related outcomes. The participants are an age-stratified sample of men from eastern Finland with a total of 2682 men (82·9 % of the eligible population) aged 42, 48, 54 or 60 years at the baseline examinations^(19)^. We excluded participants with missing data on serum *n*-6 PUFA (*n* 202) and with the history of CHD (*n* 609) from the analysis. Hence, the final analysis was conducted with 1871 men.

### Measurements

At the baseline examinations, fasting venous blood samples were collected between 08.00 and 10.00. The subjects were instructed to abstain from ingesting alcohol for 3 d and from smoking and eating for 12 h before giving the sample. Details of the other determinants, including medical history, current medications, smoking status, alcohol intake, serum lipids and resting blood pressure measurements, have been reported previously^(20)^. Physical activity was determined based on the 12-month leisure-time physical activity questionnaire and expressed as kcal/d^(21)^. BMI was computed as the ratio of weight in kilograms to the square of height in metres. Height and weight were measured by a study nurse during the study visit. Hypertension was defined as systolic/diastolic blood pressure > 140/90 mmHg or use of antihypertensive medication. Dietary intakes were assessed at the time of blood sampling in 1984–1989 with an instructed and interviewer-checked 4-d food record (three weekdays and one weekend day) by household measures^(22)^. The information of the years of education and annual income of study participants were obtained by using self-administered questionnaires.

### Serum fatty acid measurements

Serum fatty acids were measured in 1991 from samples that had been stored at –80°C in one gas chromatographic run without preseparation, as described previously^(23)^. Serum fatty acids were extracted with chloroform-methanol. The chloroform phase was evaporated and treated with sodium methoxide, which methylated esterified fatty acids. Quantification was conducted with reference standards (Check Prep Inc., Elysian, MN). Each analyte had an individual reference standard, and an internal standard was eicosan. Fatty acids were chromatographed in an NB-351 capillary column (HNU-Nordion) by a Hewlett-Packard 5890 Series II gas chromatograph (Hewlett-Packard Company, since 1999 Agilent Technologies Inc.) with a flame ionisation detector. Results were presented as a proportion of total serum fatty acids. For repeated serum fatty acid measurements, the CV was 8·7 % for LA (18:2*n*-6), 11·6 % for GLA (18:3*n*-6), 8·3 % for DGLA (20:3*n*-6) and 9·9 % for AA (20:4*n*-6). For the serum total *n*-6 PUFA concentration, we used the sum of LA, GLA, DGLA and AA.

### Exercise test and exercise electrocardiography

A maximal symptom-limited exercise stress test was conducted at the baseline examinations in 1984–1989. It was performed between 08.00 and 10.00 using an electrically braked bicycle ergometer (Medical Fitness Equipment 400 L bicycle Ergometer)^(24)^. ECG was recorded continuously with the Kone 620 electrocardiograph (Kone)^(25)^. The Mason-Likar lead system including V1, V5 and a VF lead connection was used. An ECG was recorded in 30 s intervals during exercise and at least 5 min during recovery while the subject was sitting on the bicycle. The criteria for ischaemia were horizontal ECG during exercise or down-sloping ST depression ≥ 1·0 mm at 80 ms after J point or any ST depression of > 1·0 mm at 80 ms after J point^(25)^.

### Statistical analysis

The univariate associations of the participants’ serum total *n*-6 PUFA concentration with demographic, lifestyle and clinical characteristics at baseline were assessed by means and linear regression for continuous variables and *χ* ^2^ test and Mantel-Haenszel test for categorical variables. We applied Spearman’s correlation coefficients (r) to estimate the correlations between the fatty acids.

Logistic regression models were used to estimate the OR for the occurrence of exercise-induced myocardial ischaemia in exposure quartiles, with the lowest category as the reference. Two different models were used to control for potential confounding factors. Model 1 was adjusted for age (years) and year of examination. Model 2 included the variables in model 1 plus BMI (kg/m^2^), smoking (pack-years), leisure-time physical activity (kcal/d), alcohol consumption (g/week), serum total long-chain *n*-3 PUFA concentration (DHA + docosapentaenoic acid (DPA) + EPA as a percentage of total serum fatty acids), hypertension, family history of ischaemic heart disease, intake of SFA (% of total energy intake), and vegetables, fruit and berries (g/d). All quantitative variables were entered in the models as continuous variables. The covariables in the analyses were selected based on the previous studies in the KIHD with the serum *n*-3 PUFA^(26)^ or on associations with exposures or outcomes in the present analysis. Missing values in covariates (40 in pack-years of smoking, 15 in dietary intakes, 10 in leisure-time physical activity, 8 in BMI, 3 in alcohol consumption and 1 in education) were replaced with the cohort means. For assessing the linear trends across the *n*-6 PUFA quartiles, the median value of each fatty acid quartile was used as a continuous variable. Statistical significance of the potential interactions by age, BMI, leisure-time physical activity and alcohol consumption (using the median values as the cut-off) was assessed by likelihood ratio test using a multiplicative interaction term. All *P*-values were two-tailed (*α* = 0·05). Data were analysed using the SPSS software version 27 (IBM Corp).

## Results

### Baseline characteristics

The mean (s d) age of the participants was 52·4 (5·3) years. The mean (sd) serum total *n*-6 PUFA concentration, as a percentage of all serum fatty acids, was 33·04 % (4·61). Table 1 shows the mean concentrations and correlations between individual *n*-6 and *n*-3 PUFA. The correlations were mostly weak, except for the positive correlations between GLA and DGLA and between AA and the long-chain *n*-3 PUFA. Characteristics of the participants according to quartiles of the serum total *n*-6 PUFA concentration are presented in Table 2. Men with higher serum total *n*-6 PUFA concentration were younger, more educated, more physically active and less likely to have hypertension or diabetes, or to smoke. They also had higher ALA and fruit, berry, and vegetable consumption, lower alcohol and SFA intake, lower BMI and lower serum TAG and higher HDL-cholesterol concentrations.

**Table 1.** Mean values of serum *n*-6 and *n*-3 PUFA and Spearman’s correlation coefficients (Mean values and standard deviations)

	LA (%)	GLA (%)	DGLA (%)	AA (%)	ALA (%)	EPA (%)	DPA (%)	DHA (%)
Mean	26·62	0·28	1·34	4·81	0·74	1·67	0·55	2·46
sd	4·43	0·11	0·27	1·01	0·24	0·91	0·10	0·73
Correlations								
LA	1	–0·20*	–0·09*	0·11*	0·22*	–0·18*	–0·09*	–0·03
GLA		1	0·54*	0·19*	–0·07*	0·06*	0·08*	–0·19*
DGLA			1	0·08*	–0·12*	–0·15*	–0·08*	–0·25*
AA				1	–0·38*	0·44*	0·34*	0·44*

LA, linoleic acid; GLA, *γ*-linolenic acid; DGLA, dihomo-*γ*-linolenic acid; AA, arachidonic acid; ALA, *α*-linolenic acid; DPA, docosapentaenoic acid.

* *P*-value < 0·05.

**Table 2.** Baseline characteristics according to the quartiles of total serum *n*-6 PUFA concentrations* (Mean values and standard deviations; percentages)

	Serum total *n*-6 PUFA quartile (%)												
	Q1 (<30·01) (*n* 467)			Q2 (30·01–33·21) (*n* 468)			Q3 (33·22–36·05) (*n* 468)			Q4 (>36·05) (*n* 468)			
Variables	Mean	sd	%	Mean	sd	%	Mean	sd	%	Mean	sd	%	*P*_for trend_
Age (years)	52·9	5·0		52·7	5·2		52·4	5·3		51·6	5·8		<0·001
Educations (years)	8·7	3·3		8·5	3·3		9·1	3·8		9·5	3·8		<0·001
BMI (kg/m^2^)	28·2	4·0		27·2	3·4		26·1	2·9		25·6	2·8		<0·001
Physical activity (kcal/d)	124	169		130	151		133	149		164	201		<0·001
Current smoker (%)			37·0			28·6			30·6			23·3	<0·001
Alcohol consumption (g/week)	101	158		75	111		63	90		55	89		<0·001
Diabetes (%)			7·5			5·1			2·8			2·8	0·001
Hypertension (%)			68·5			59·2			49·8			47·2	<0·001
Medications (%)†			22·9			14·7			12·2			10·0	<0·001
Dietary total fat intake (E%)	38·04	6·21		38·86	5·89		38·84	5·55		38·65	5·50		0·08
Dietary SFA (E%)	18·41	4·31		18·62	4·10		18·16	3·67		17·25	3·44		<0·001
Dietary ALA intake (E%)	0·52	0·23		0·54	0·21		0·60	0·22		0·67	0·20		<0·001
Dietary EPA intake (E%)	0·06	0·08		0·06	0·07		0·06	0·06		0·05	0·05		0·36
Dietary DHA intake (E%)	0·11	0·14		0·10	0·14		0·10	0·12		0·09	0·10		0·03
Dietary LA intake (E%)	2·90	1·24		3·08	1·23		3·33	1·18		3·92	1·26		<0·001
Dietary AA intake (E%)	0·07	0·03		0·07	0·03		0·07	0·03		0·07	0·03		<0·001
Fruit, berries and vegetables (g/d)	238	159		253	147		265	161		281	159		<0·001
Energy intake (kcal/d)	2418	626		2453	660		2459	568		2497	613		0·06
Serum TAG (mmol/l)	1·86	1·05		1·26	0·53		1·06	0·45		0·88	0·36		<0·001
Serum HDL-cholesterol (mmol/l)	1·20	0·27		1·28	0·27		1·34	0·29		1·39	0·30		<0·001
Serum LDL-cholesterol (mmol/l)	3·90	0·98		4·05	0·95		4·07	0·97		3·99	1·02		0·19
Blood glucose (mmol/l)	4·99	1·35		4·71	0·79		4·62	0·71		4·55	0·79		<0·001
Serum total long-chain *n*-3 PUFA (%)‡	4·70	1·95		4·79	1·65		4·77	1·45		4·45	1·20		0·02
Serum LA (%)	21·16	2·42		25·28	1·34		28·01	1·33		32·00	2·5		<0·001
Serum GLA (%)	0·31	0·12		0·28	0·10		0·28	0·10		0·27	0·10		<0·001
Serum DGLA (%)	1·33	0·26		1·35	0·27		1·34	0·27		1·33	0·26		0·81
Serum AA (%)	4·32	1·00		4·82	0·97		4·97	0·94		5·13	0·95		<0·001

Q; quartile; E%, percent of energy intake; ALA, *α*-linolenic acid; LA, linoleic acid; AA, arachidonic acid; GLA, *γ*-linolenic acid; DGLA, dihomo-*γ*-linolenic acid.

* Values are mean (sd) or median (interquartile range, IQR) for continuous variables and *n* (%) for categorical data. Data were analysed with analysis of variance and linear regression for continuous variables and *χ* ^2^ test and Mantel-Haenszel test for categorical variables.

† Antihypertensive and anti-hyperlipidemic medication.

‡ Serum total long-chain *n*-3 PUFA was the sum of EPA, docosapentaenoic acid (DPA) and DHA.

### The serum *n*-6 PUFA concentrations and odds of exercise-induced myocardial ischaemia

**Fig. 1.**
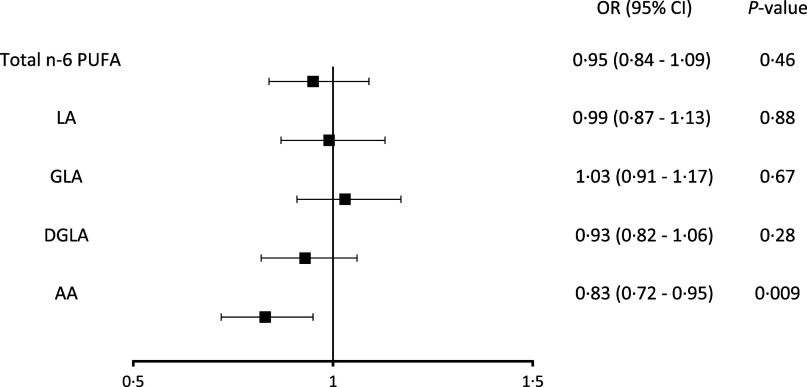
Multivariable-adjusted odds of exercise-induced myocardial ischaemia for 1 sd change in the serum *n*-6 PUFA. LA, linoleic acid; GLA, *γ*-linolenic acid; DGLA, dihomo-*γ*-linolenic acid; AA, arachidonic acid

Exercise-induced myocardial ischaemia was found in 332 (17·7 %) of the 1871 men. Serum total *n*-6 PUFA concentration was not associated with myocardial ischaemia during exercise (Table 3). Among the individual fatty acids, only serum AA concentration was associated with the odds for exercise-induced myocardial ischaemia. After multivariable adjustments, those in the highest *v*. the lowest serum AA concentration quartile had 50 % lower odds for exercise-induced myocardial ischaemia (OR 0·50, 95 % CI 0·34, 0·76, *P*-trend across quartiles < 0·001) (model 2, Table 2). Each 1-sd increase in the serum AA concentration was associated with 17 % lower odds (OR 0·83, 95 % CI 0·72, 0·95) (Fig. 1). LA, GLA and DGLA were not associated with exercise-induced myocardial ischaemia (Table 3, Fig. 1). Age, physical activity, alcohol consumption or BMI did not modify the associations (*P*-values for interactions were > 0·73 for LA, > 0·13 for GLA, > 0·28 for DGLA and > 0·62 for AA).

**Table 3.** OR for exercise-induced myocardial ischaemia in quartiles of serum *n*-6 PUFA* (Odds ratios and 95 % confidence intervals)

	Exposure quartile							
	Q1 (*n* 467)	Q2 (*n* 468)		Q3 (*n* 468)		Q4 (*n* 468)		*P*_for trend_
		OR	95 % CI	OR	95 % CI	OR	95 % CI	
Total *n*-6 PUFA (%)	<30·01	30·01–33·21		33·22–36·05		>36·05		
Number of events	87	89		83		73		
Model 1†	1 (reference group)	1·03	0·74, 1·43	0·95	0·68, 1·33	0·86	0·61, 1·22	0·38
Model 2‡	1 (reference group)	1·02	0·73, 1·44	0·98	0·69, 1·39	0·86	0·59, 1·24	0·43
Linoleic acid (%)	<23·68	23·68–26·65		26·66–29·52		>29·52		
Number of events	82	89		83		78		
Model 1	1 (reference group)	1·11	0·79, 1·55	1·03	0·73, 1·44	1·00	0·71, 1·42	0·94
Model 2	1 (reference group)	1·13	0·80, 1·59	1·02	0·71, 1·45	1·00	0·69, 1·46	0·89
GLA (%)	<0·21	0·21–0·26		0·27–0·35		>0·35		
Number of events	76	78		102		76		
Model 1	1 (reference group)	1·02	0·72, 1·45	1·49	1·07, 2·08	1·09	0·76, 1·54	0·33
Model 2	1 (reference group)	1·03	0·73, 1·64	1·49	1·06, 2·08	1·07	0·75, 1·53	0·40
DGLA (%)	<1·15	1·15–1·32		1·33–1·50		>1·50		
Number of events	86	84		93		69		
Model 1	1 (reference group)	0·99	0·71, 1·38	1·11	0·80, 1·54	0·80	0·57, 1·14	0·31
Model 2	1 (reference group)	0·95	0·68, 1·34	1·05	0·75, 1·48	0·74	0·51, 1·07	0·16
AA (%)	<4·12	4·12–4·74		4·75–5·44		>5·44		
Number of events	97	97		81		57		
Model 1	1 (reference group)	1·00	0·72, 1·37	0·82	0·59, 1·15	0·55	0·38, 0·78	<0·001
Model 2	1 (reference group)	1·00	0·72, 1·38	0·82	0·58, 1·16	0·50	0·34, 0·76	<0·001

Q, quartile; GLA, *γ*-linolenic acid; DGLA, dihomo-*γ*-linolenic acid; AA, arachidonic acid.

* Values are means (95 % CI).

† Model 1: adjusted for age and examination year.

‡ Model 2: adjusted for model 1 + BMI, smoking status, leisure-time physical activity, alcohol intake, intake of SFA, fruits, berries and vegetables, serum total long-chain *n*-3 PUFA concentrations, family history of ischaemic heart disease and hypertension.

## Discussion

In the present study among middle-aged and older men from eastern Finland, higher serum AA concentration was associated with lower odds of exercise-induced myocardial ischaemia, whereas the other *n*-6 PUFA, LA, GLA and DGLA had no association. To the best of our knowledge, the present study is the first one to evaluate the associations of the serum *n*-6 PUFA concentrations with exercise-induced myocardial ischaemia, so we are not able to compare our findings to other studies.

The findings in some dietary fat modification trials from the 1960s and 1970s suggested that very high LA intake could have unfavourable cardiovascular effects. However, these trials had many limitations, such as high dropout rate, short duration, low number of participants and possible high *trans*-fatty acid intake^(27)^. Also, the LA intake in those trials was much higher than in typical Western diets or when compared with the dietary recommendations^(27)^, and the fat sources with only *n*-6 PUFA used in some trials may have replaced fat sources with *n*-3 PUFA, thereby reducing *n*-3 PUFA intake^(27)^. Indeed, there is some evidence that only the trials that used LA sources that included also *n*-3 PUFA reported some benefits, whereas trials that used fat sources with only *n*-6 PUFA did not^(28)^. In contrast, for example, country-level comparisons have shown that LA content in the body was much lower in a region with high CHD mortality (eastern Finland) compared with a region with low CHD mortality (Italy)^(29)^. In addition, cohort studies have consistently suggested an inverse association of higher exposure to LA with risk of CVD^(11,12)^, and also short-term dietary trials have shown benefits on serum lipid profile^(30)^, without having harmful effects on, for example, inflammation^(31)^. Also in the current study population, higher serum LA concentrations have been associated with lower risk of CVD^(18,32)^, although the findings from the current study suggest that the associations are not explained by effects on exercise-induced myocardial ischaemia.

Less is known of the cardiovascular effects of the minor *n*-6 PUFA GLA and DGLA. GLA and DGLA and its metabolites, including PGE1, have beneficial effects on reducing inflammation and improving cardiac function^(33,34)^. In addition, in one study from the early 1980s, DGLA had an even stronger beneficial association with CHD than LA had^(35)^. However, the findings in the current study are in line with our previous results in this same cohort, where serum GLA or DGLA concentrations have not been associated with risk of CVD outcomes^(18,32)^.

Various metabolites from multiple metabolic pathways of AA in the body, including PGE2, are related to the development of myocardial ischaemia and ischaemic reperfusion injury, but the relationships between these various pathways are unclear^(36)^. Moreover, AA is a ligand for both PPAR *α* and γ that have different characteristics. PPAR*α* exhibits anti-inflammatory characteristics and preventive effects on vascular damage, while PPARγ has pro-inflammatory and atherosclerotic effects^(37,38)^. On the other hand, several AA-derived eicosanoids and metabolites may have an anti-inflammatory role^(39)^, vasodilatory effects and vascular healing role to prevent ischaemic heart disease and CVD^(40)^. From different AA metabolites, PGD2^(41)^, PGI2^(42)^ and epoxyeicosatrienoic acids (EET)^(43)^ have potentially protective effects on cardiac ischaemia/reperfusion injury. In addition, the potent anti-inflammatory metabolite lipoxin A4, derived from AA, increases in the body during exercise^(44)^, thereby exerting cardioprotective effects through the inhibition of PGE1 and leukotriene production, as well as by reducing other plasma inflammatory component concentrations, such as IL-6 and TNF-*α*^(44–46)^. The role of AA as a precursor for multiple cardioprotective eicosanoids provides one plausible explanation for the specific protective of AA.

In addition, exercise can change the concentrations of *n*-6 PUFA metabolites and increase those with protective effects. Giordano *et al.* found an elevation of plasma EET and dihydroxyeicosatrienoic acid (DHET) during a bicycle ergometer exercise test on fourteen healthy participants^(47)^. Similarly, a study on six healthy subjects aged 23–53 years in Germany showed that during a maximal treadmill test, the plasma metabolites of AA in cytochromes P450 pathway, including EET and DHET, increased, while the concentration of lipoxygenase-dependent metabolites of AA with pro-inflammatory and vasoconstriction characteristics (thromboxane A, thromboxane B, etc.) were not changed^(48)^. EET can affect the vasodilation and activation of Ca-activated K+ channels (BKca) in vascular cells, and DHET can affect the haemodynamics and responses of the cardiovascular system^(48,49)^. In addition, an experimental study found that EET to DHET ratio can modulate vascular tone due to inducing the release of endothelial nitric oxide; this ratio can increase during the exercise test^(48,49)^. Therefore, the exercise test, as a short-term exercise that increases haemodynamics like heart rate and blood pressure, might elevate the heart-protective AA metabolites^(48)^, which may partially explain the lower probability of myocardial ischaemia during an exercise test among those with higher serum AA in our study.

The cardioprotective effects of the *n*-3 PUFA and their metabolites (three-series PG and five-series leukotrienes) have long been recognised^(50)^. However, despite the concern about possible pro-inflammatory effects of the *n*-6 PUFA, especially AA, recent research has shown that some *n*-6 PUFA metabolites (like PGI2 and lipoxin A4 from AA) can have cardioprotective effects similar to the *n*-3 PUFA^(11)^. Furthermore, there is enzymatic competition between EPA and AA for synthesis of some metabolites^(51)^; however, sufficient amounts of AA and EPA are needed for their beneficial functions in cardiovascular health^(52)^. For instance, although higher EPA is associated with lower pro-inflammatory eicosanoids from AA^(51)^, AA provokes the metabolic pathway to increase PGI3 synthesis from EPA^(53)^. Therefore, adequate levels of these fatty acids may help prevent atherosclerosis and CHD^(52)^. In our study, there was a positive correlation between AA and the long-chain *n*-3 PUFA, which could indicate that the associations observed with AA could be due to the long-chain *n*-3 PUFA. However, adjustment for the long-chain *n*-3 PUFA concentrations did not attenuate the inverse association of AA with exercise-induced myocardial ischaemia.

Our current study had several strengths. The study included a large sample size with information from an exercise test with ECG measurements. The use of serum *n*-6 PUFA measurements instead of assessing dietary intakes reduces the bias of misreporting and misclassification that can introduce random error and attenuate associations. In this cohort, serum LA and AA concentrations both associate with their dietary intakes^(18)^. However, the concentrations are known to be influenced also by genetic factors that can also have an impact on the association of the *n*-6 PUFA with CVD risk^(12)^. For instance, polymorphisms of the *FADS1* gene do not only regulate the *n*-6 PUFA concentrations but are also linked to, for example, heart rate and some CVD outcomes^(12,54,55)^. Unfortunately, we do not have data on such genetic factors.

The extensive database from the KIHD enabled examination of potential confounders. One potential limitation of our study was that the participants were middle-aged and older men from eastern Finland, so the findings may not be generalisable to other populations, age groups or to women. Another limitation related to the cross-sectional study design is that we cannot derive information on causal relationships between the serum *n*-6 PUFA concentrations and exercise-induced myocardial ischaemia. Moreover, in contrast to the *n*-6 PUFA in adipose tissue and erythrocyte fatty acid measurements that reflect long-term intakes, our data were obtained from serum fatty acids, which reflect only more recent intake^(56)^. However, all the baseline serum *n*-6 PUFA concentrations in this study cohort correlate reasonably well with repeated measurements several years apart (correlation coefficients ≥ 0·5)^(18)^, suggesting that they reflect typical concentrations. Finally, we did not have data on plasma levels of cytokines and prostanoids to investigate the associations of *n*-6 and *n*-3 PUFA with these particles to understand the full potential and beneficial/harmful actions of the PUFA.

In conclusion, higher serum AA concentration was associated with lower occurrence of exercise-induced myocardial ischaemia among middle-aged and older men. This finding may provide one explanation for the previously observed inverse association of AA with risk of CHD and CVD. However, no association was found with the other *n*-6 PUFA in the current study. Additional large-scale studies in different populations of different ages and sexes are needed to confirm our findings and to provide knowledge of the mechanisms of the *n*-6 PUFA on myocardial ischaemia.
